# The Effects of Replacing Soybean Meal with *Chlorella vulgaris* in Laying Hen Diets on Performance and Physical Characteristics of Eggs

**DOI:** 10.3390/ani14172552

**Published:** 2024-09-02

**Authors:** Obete Madacussengua, Ana Rita Mendes, Cátia Falcão Martins, Daniela Carvalho, André Martinho de Almeida, Madalena Lordelo

**Affiliations:** 1 LEAF-Linking Landscape, Environment, Agriculture and Food Research Center, Instituto Superior de Agronomia, Universidade de Lisboa, Tapada da Ajuda, 1349-017 Lisbon, Portugal; omadacussengua@isa.ulisboa.pt (O.M.); rmendes@isa.ulisboa.pt (A.R.M.); catiamartins@isa.ulisboa.pt (C.F.M.); danielacarvalho@isa.ulisboa.pt (D.C.); aalmeida@isa.ulisboa.pt (A.M.d.A.); 2 Associate Laboratory TERRA, Instituto Superior de Agronomia, Universidade de Lisboa, Tapada da Ajuda, 1349-017 Lisbon, Portugal

**Keywords:** egg quality, laying hens, microalgae, *Chlorella vulgaris*

## Abstract

**Simple Summary:**

The use of alternative feed sources such as *Chlorella vulgaris* in diets for birds, particularly for laying hens, has been gaining attention due to its potential benefits. This study evaluates how different dietary levels of *Chlorella vulgaris* affect the productive performance and physical quality of eggs from laying hens. The results indicated that the incorporation of *Chlorella vulgaris* in the diets of the hens had a limited impact on performance parameters and positively influenced egg quality and yolk color.

**Abstract:**

*Chlorella vulgaris* (CV) is a microalga with considerable nutritional value, containing high levels of protein, carotenoids, and polyunsaturated fatty acids, which have the potential to positively influence the productive performance and egg quality of laying hens. CV emerges as a more sustainable ingredient than soybean meal (SBM) as it can be produced locally and with fewer inputs. In this regard, a study was conducted with 48 H&N Brown Nick strain laying hens, at 19 weeks of age, over a period of 16 weeks. The hens were divided into four treatments, with 12 replicates each. The treatments consisted of providing four different diets: a control diet based on corn and SBM without the inclusion of CV, and three other diets with partial substitution of SBM by 2.5, 5, and 10% of CV. The results showed that the inclusion of CV in the diets did not significantly affect feed intake, feed conversion ratio, or egg production (*p* > 0.05). In addition, moderate CV levels increased egg weight, while higher levels reduced it. Haugh units, yolk index, albumen index, egg surface area, specific density, and translucency were not affected (*p* > 0.05), while shell index and shell thickness were lower in the groups that received the CV (*p* < 0.0001). Yolk color improved significantly with increasing CV levels (*p* < 0.0001), with darker, more intensely colored yolks at higher CV concentrations. The results of this study suggest that the incorporation of CV in the diet of laying hens had a limited impact on performance parameters. In addition, CV supplementation can positively influence egg quality and yolk color, although careful consideration of optimal levels is necessary to avoid negative effects on other parameters.

## 1. Introduction

Continuing population growth, increased income levels, and greater nutrition awareness drive the demand for animal proteins, such as meat and eggs. As a result, there is an increasing need for corn and soybean meal (SBM), which are the two main conventional ingredients used in poultry feeding [1,2]. This situation presents a particular challenge as the European Union heavily relies on the importation of these feedstuffs. In addition to these challenges, Europe also faces restrictions on the use of antibiotics in animal feeding. This is due to concerns about factors such as increased antibiotic resistance in humans and animals, the presence of antibiotic residues, and the reduction of natural gut microflora [3,4]. These restrictions, coupled with the recent Eurozone inflation, due to continuing geopolitical conflicts, significantly increase production costs and ultimately lead to higher prices of meat and eggs in the market.

Therefore, to face these scenarios, in recent years, the use of alternative feed ingredients in poultry farming has become an increasingly important trend not only for conventional ingredients and antibiotics but also for pigments [5,6,7,8]. It is important to incorporate new sources of energy, high-quality proteins and lipids into poultry diets, especially in laying hens, in order to ensure a higher production rate and better-quality eggs. In addition, eggs enriched with certain nutrients through dietary manipulation can significantly contribute to health benefits for consumers, by reducing the incidence of chronic diseases, such as cancer and obesity [9,10].

*Chlorella vulgaris* (CV), a high-quality microalga, has been studied for its potential as an alternative to conventional feed ingredients, antibiotics, and synthetic pigments in poultry diets. This is mainly due to CV’s notable nutritional profile, which includes high levels of crude protein, carotenoids, polyunsaturated fatty acids, vitamins, and minerals [11,12,13]. Incorporating CV into the diets of laying hens has shown promising results in terms of improving productive performance, egg quality, and immunological function. This suggests that CV can be a valuable ingredient for enhancing the overall health and productivity of poultry [13,14]. Additionally, CV has the potential to be a more sustainable ingredient than SBM, as it does not need a large amount of arable land, and it can be produced locally and with fewer inputs, since it only requires sunlight, and inorganic compounds such as nitrogen, phosphorus and potassium, and water [15,16]. The inclusion of CV in the diet of laying hens can have beneficial effects, such as reducing total cholesterol in the yolk, decreasing concentrations of saturated fatty acids, and increasing levels of omega-3 polyunsaturated fatty acids, mainly linoleic acid and arachidonic acid [17,18]. Furthermore, hens supplemented with CV produce eggs with more orange yolks [19]. These characteristics may turn eggs from chickens fed with this microalga into a healthier and more appreciated option for consumers [13,20].

Regarding low levels of CV incorporation in the diet of laying hens, Halle et al. [21] observed that the inclusion of 0.25, 0.5, and 0.75% of CV in the feed of laying hens aged 22 weeks, for 28 weeks, had no impact on the laying rate, egg weight, and feed conversion ratio (FCR), but resulted in more orange yolks. Zheng et al. [13] observed that when 1 and 2% of CV were added to the diet of 80-week-old laying hens for 6 weeks, egg production, yolk color, and Haugh unit showed a linear increase with higher levels of incorporation. Kor and Mohamadi [18] observed that the inclusion of 100, 200, and 400 ppm of CV in the drinking water of 63-week-old laying hens for 9 weeks did not affect egg production, egg weight, feed intake, and feed conversion ratio.

At higher incorporation levels, Kim and Kang [22] demonstrated that the addition of 5 and 7.5% of CV to the feed of 28-week-old laying hens, for 8 weeks, improved egg production, increased feed intake (FI), and also resulted in more orange yolks. In the same line, Grigorova [23] observed that including 10% of *Chlorella genus* in the feed of older (74 weeks) laying hens for 5 weeks resulted in positive effects on egg weight, eggshell thickness, and yolk color intensity. In a study by Lipstein, Hurwitz, and Bornstein [20], it was shown that introducing 12% *Chlorella* into the diet of laying hens for 2 weeks did not result in any significant changes in body weight (BW), FI, FCR, egg production, and eggshell quality, except for alterations in yolk pigmentation.

While replacing soybean meal with CV in laying hens’ diets offers potential benefits, it presents a financial challenge due to the significant difference in production scale. Global soybean production for the 2023/2024 season is estimated at 399 million tons, a 6.3% increase from the previous year [24], whereas *Chlorella* sp. production currently sits at only 20,000 tons annually [25]. This disparity in production leads to a higher price for CV compared to soybeans. Nevertheless, CV’s potential for local production and reduced input requirements make it a promising alternative to soy in laying hen diets, despite the current price differential.

Despite growing interest in utilizing CV as a feed supplement, the long-term effects of high CV incorporation (above 2.5%) in poultry diets remain largely unexplored. Moreover, incorporating high doses of CV (10%) presents a promising alternative to soybean meal, offering a high-protein source that can potentially replace the primary protein in laying hen diets. Therefore, the objective of the current work is to assess the effects of replacing SBM with the incorporation of CV into the diet of laying hens for 16 weeks, specifically focusing on productive performance and egg physical characteristics. This research is particularly significant in the current context, as the conservation of natural resources is paramount for mitigating climate change and establishing a sustainable supply of feed ingredients for the livestock industry.

## 2. Materials and Methods

### 2.1. Experimental Birds and Management

Forty-eight 19-week-old commercial laying hens of the H&N Brown Nick strain were individually housed in cages measuring 1.1 m in length, 0.5 m in width, and 1.3 m in height for a duration of 16 weeks. The hens were kept in a controlled environment with standardized conditions for both sanitation and temperature. They were provided with 15 h of daily lighting, fed ad libitum and had unlimited access to water. Thermo-hygrometers (Pur Line, WSO1N, Climacity, S.L., Madrid, Spain) were utilized to monitor the temperature and relative humidity within the cages. The laying hens were sourced from the commercial farm Clara & Gema (Barreira d’água, Leiria, Portugal).

The 48 laying hens were divided into four groups, each consisting of 12 replicates. The treatments included a control group that received a diet based on corn and SBM without the inclusion of CV, as well as three other groups that received diets with partial replacement of SBM with 2.5, 5, and 10% of CV, respectively. The diets were formulated to meet the nutritional requirements of the laying hens, following the recommended guidelines for commercial production of the H&N Brown Nick hens (Table 1). The nutritional analysis of the experimental diets was carried out following the guidelines set by the Association of Official Analytical Chemists [26] and the results are in Table 1 and Table 2. All trial procedures were duly approved by the Animal Welfare Committee of Instituto Superior de Agronomia of the University of Lisbon and the Portuguese authority, Direção-Geral da Alimentação e Veterinária (DGAV).

### 2.2. Microalgal Biomass

*Chlorella vulgaris* was sourced from Allmicroalgae-Natural Products S.A (Pataias, Portugal), a national company committed to sustainable practices. The algae is cultivated using a natural, autotrophic process, harnessing sunlight and CO_2_ to produce oxygen. After harvesting, the concentrated biomass undergoes centrifugation to separate the liquid from the algae, followed by spray drying to produce a stable, high-quality product [27].

### 2.3. Productive Performance of Laying Hens

During the experimental period, data related to productive performance was collected and recorded. This included daily FI, egg production, and egg weight. Body weight was monitored every week, and FCR was calculated on a weekly basis. Additionally, weekly measurements of egg weight were taken, excluding broken and soft-shelled eggs. These recorded measurements allowed for a comprehensive analysis of the hen’s productivity throughout the experimental period. When calculating the feed conversion index, we consider the amount of food consumed in relation to the weight of the eggs produced. A lower FCR value indicates a more efficient conversion of food into eggs. This means that less food is needed to produce a given weight of eggs, making the production process more efficient.

### 2.4. Egg Physical Parameters

Three eggs per treatment were collected twice a week and analyzed for each experimental treatment, resulting in a total of 288 eggs over 12 consecutive weeks. The analysis protocol consisted of several steps. Initially, each egg was individually weighed to determine its mass, candled to measure the height of the air cell, and subsequently inspected for any cracks. A caliper was then utilized to measure the height and equatorial diameter of the eggs. After initial measurements, eggs were broken on a flat surface to separate the yolk and albumen. The width and length of the albumen as well as the diameter of the yolk were measured using a caliper. The height of the thick albumen and the yolk was determined using a tripod micrometer.

To determine the color of the yolk, it was used a Nix Pro 2 Color Sensor colorimeter (Nix Sensor Ltd., Hamilton, ON, Canada). This colorimeter measured the yolk color using the CIELAB system, providing precise information on the luminosity (L*), the red/green color component (a*), and the yellow/blue color component (b*) of the yolk. In addition, measurements of the pH of the egg yolk and albumen were performed using a potentiometer (Metrohm 744, Metrohm AG, Herisau, Switzerland). A visual observation was also carried out to identify the presence of blood and meat spots in the yolk and albumen.

To evaluate the proportion of the shell in relation to the rest of the egg, the eggshells were dried in an oven at 60 °C for 48 h to calculate the percentage of shell weight in relation to the total weight of the egg.

For specific gravity analysis, the eggs were subjected to saline solutions with densities ranging from 1.065 to 1.095, with incremental concentrations of 0.005, following the procedures proposed by Butcher and Miles [28].

**Figure 1:**
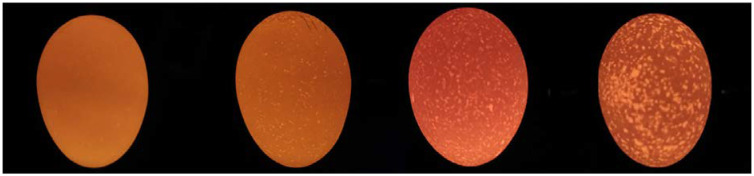
Four reference samples used for the scoring method, showing scores [1], [2], [3], and [4] from left to right. Source: Wang [29].

For translucency analysis, 100 eggs from each group were stored at 18 degrees. On the fifth day of storage, the eggs were individually classified into 4 levels of scoring (Figure 1) according to the scale presented by Wang [29]. This scale allowed the eggs to be classified into four levels of translucency: (1) absence of translucent spots; (2) few and small translucent spots; (3) more translucent spots widely distributed on the eggshell; and (4) presence of many larger spots.

Based on the results of physical measurements, the following parameters were calculated: (1) Haugh unit was determined using the equation developed by Haugh [30]: log (albumen height—1.7 × egg weight 0.37) × 100. (2) Egg surface area was determined using the equation proposed by Thomson [31]: 4.67 (Egg weight) 2/3. (3) The shape index was calculated by dividing the equator diameter by the egg height and multiplying the result by 100, as proposed by Khalafalla [32]. (4) The yolk index was determined by applying the equation presented by Saki [33]: (yolk height/yolk diameter) × 100. (5) The albumen index was determined by dividing the height of the thick albumen by its equatorial width (https://www.sciencedirect.com/science/article/pii/S0032579119554311, accessed on 4 August 2024). (6) The eggshell index was calculated by dividing the shell weight by the shell surface and multiplying the result by 100 [34]. (7) Shell thickness was calculated by dividing the eggshell index by 23.5 [34].

### 2.5. Statistical Analysis

Data analysis was performed using the SAS software 9.4 package (SAS Inst. Inc., Cary, NC, USA). Multivariate tests of the general linear and quadratic model (GLM) were conducted to examine the impact of dietary treatment as a single effect. The dietary treatment was considered a fixed effect in all statistical models. Tukey’s test was employed to identify significant differences between the means, with a level of statistical significance set at *p* < 0.05.

## 3. Results

### 3.1. Production Performance

The effects of adding CV on the performance parameters of laying hens over a 16-week period are summarized in Table 3. Overall, no significant differences were observed in initial body weight, final body weight, egg production, egg weight, feed conversion ratio, and feed intake between the control group and the groups that received CV (*p* > 0.05). Additionally, no mortality was recorded during the trial. The feed conversion rate in all groups remained below 2.08, which is the maximum recommended by the H&N strain manual guidelines [35]. It was noted that the average daily feed intake exceeded the recommended amount of 118 g/day in all groups, except for the group that received 10% CV, although there were no significant differences between the groups. The *p*-values for linear and quadratic trends were both significant for egg weight, suggesting a treatment effect on this parameter with moderate CV levels increasing egg weight and higher levels reducing it.

### 3.2. Physical Characteristics of Eggs

The results of the physical characteristics of the egg are presented in Table 4. According to the classification by Akouango [36], eggs with Haugh units above 70 are considered excellent in terms of quality. Eggs with Haugh units between 70 and 60 are considered acceptable, while eggs with Haugh units below 60 are considered to be of poor quality. Based on our results, the average Haugh unit values were higher than 70, suggesting that the eggs can be classified as excellent in terms of quality (Table 4). Also, according to Table 4, the eggs produced by the hens that received 10% CV showed a significantly higher value for Haugh units compared to the other CV groups, but not in relation to the control group (*p* = 0.0003). Both linear and quadratic trends were significant for Haugh units.

Regarding the surface area of the eggs, a measurement that predicts the weight and hatchability of chicks, as well as the characteristics of the eggshells [37,38], no significant differences were found between the control group and the groups that received microalgae (*p* = 0.00080, Table 4).

The shape index is a measure used to determine whether eggs are normal, sharp, or rounded in shape. According to Altuntaş and Şekeroǧlu [39], values between 72% and 76% indicate a normal egg shape. Values below this range suggest a sharp shape, while values above indicate a round shape. Based on our results, the average values obtained for the shape index were higher than 76%; therefore, they were considered round-shaped eggs (Table 4). The shape index of the eggs from the hens that received 5% and 10% of CV was significantly higher compared to the eggs from the control group (*p* < 0.0001). Moreover, the linear and quadratic effects were significant for shape index, with an increase in CV associated with an increase in the shape index.

According to Sharp and Powell [40], high-quality eggs typically have a yolk index value ranging from 30 to 50%. Based on the results of our study, the eggs can be considered of high quality, as their yolk index values range from 47.8 to 49.8% (Table 4). However, no significant differences were found in the yolk index values between the groups (*p* = 0.114).

According to Table 4, there was a significant linear trend, with highest levels of CV increasing the albumen index. Eggs from the CV 5% experimental group had a significantly lower albumen index compared to the eggs from the CV 2.5% and CV 10% experimental groups (*p* = 0.017).

Eggshell quality is one of the most significant factors that impact the poultry industry, with significant economic implications for both egg production and hatchability. In our study (Table 4), the incorporation of CV had a negative effect on the shell index and shell thickness (*p* < 0.0001). The control group had the highest shell index and shell thickness, with both linear and quadratic trends being significant (Table 4).

Another important parameter in determining egg quality is the specific gravity, which represents the ratio of shell weight to the weight of other components in the egg. Additionally, it serves as an indicator of the eggs’ susceptibility to cracking during processing [28]. In our study, the inclusion of CV in the hens’ diet did not have a significant effect on the specific gravity of the eggs (*p* = 0.9955), as indicated by the results presented in Table 4.

The pH of the albumen and yolk is one of the best indicators of egg freshness. The pH levels are generally unaffected by age or breed, but are affected by diet and storage time. The pH values presented in Table 4 indicate that there are no significant differences between the treatments (*p* > 0.05).

In our study (Table 5), we observed a significant enhancement in yolk color scores when different doses of CV were added to the feed of laying hens, compared to the control group (*p* < 0.0001). The incorporation of CV in the diet led to an increase in both the redness (a*) and yellowness (b*) of the egg yolk, indicating the transfer of microalgal carotenoids to the yolk (*p* < 0.0001). As the amount of CV added to feed increased, we noticed a shift in yolk color from a yellow to a more vibrant orange shade. Additionally, the L* (lightness), b* (yellowness), and a* (redness) values all exhibited significant linear and quadratic trends (*p* < 0.0001), with CV leading to darker (lower L*), more yellow (higher b*), and more red (higher a*) yolks as the CV level increased.

Regarding translucency, according to the results presented in Table 6, at the end of the five-day preservation period, a significant number remained at score 1, while a considerably reduced number managed to reach score 4. Overall, these results indicate that the diet did not have an influence on the translucency of the eggshell, as indicated by similar scores across all treatment groups. The scores for each level of translucency remained consistent regardless of the CV concentration in the diet.

## 4. Discussion

The exceptional nutritional profile of *Chlorella*, containing proteins, lipids, vitamins, and carotenoids, suggests that incorporating it into animals’ diets could enhance final body weight, egg production, and egg weight [19]. However, the present study did not observe this improvement, possibly due to the microalgae’s recalcitrant cell wall, along with abundant polysaccharides and phenolic compounds, which might impede nutrient digestibility [7,41,42]. Conversely, the higher apparent feed intake in the control group may stem from the birds’ familiarity with that diet, while the challenge of accepting the microalgae-containing diet, particularly in the initial trial days, could be attributed to its strong color and aroma.

Overall, our findings align with those of Lipstein [20], who reported that incorporating 12% CV into laying hens’ diets did not significantly impact egg production and feed conversion rate. Consistent with our results, Kim and Kang [22] also found that the feed conversion rate remained unaffected by the inclusion of algae. Nevertheless, Kim and Kang [22] observed improvements in egg production rate and feed intake with the addition of 5% and 7.5% CV to the hens’ diet.

Concerning egg weight, our current data suggest that the treatments had a noticeable effect on egg weight. The most striking finding is the significant impact on egg weight, particularly with the inclusion of 2.5% of CV. These results could imply that the treatments had a specific effect on egg weight rather than general growth or production metrics. Interestingly, Grigorova [23] noted a notable increase in egg weight when 10% *Chlorella genus* was included in the diet of laying hens.

Studies investigating the inclusion of low concentrations of CV in the diet of laying hens have similarly shown inconsistent results. For instance, Halle et al. [21] noted that adding 0.25, 0.5, and 0.75% of CV to the diet of laying hens did not affect laying rate, egg weight, and feed conversion. On the other hand, Zheng et al. [13] demonstrated a significant improvement in egg production with the inclusion of 1% and 2% of CV, although no significant differences were observed in egg weight and consumption.

Studies involving the use of other microalgae, such as *Nannochloropsis gaditana*, *Nannochloropsis oceanica*, and *Spirulina platensis*, have shown contradictory results in terms of productive performance, as reported by Abbas et al. [43], Omri et al. [37], and Tufarelli et al. [44]. The discrepancies in the results may be due to challenges in accurately determining the doses of microalgae added to the diets, issues related to the housing conditions of the animals, the use of different bird strains in the studies, variations in the age of the animals, differences in the composition of the diet, and various management systems used [45,46]. In addition, the abundance and composition of microalgae are influenced by several factors, including species, temperature, pH, nutrient availability, and light exposure. These variations in production conditions can lead to differences in the nutritional composition of microalgae, ultimately contributing to the variability in experimental results when incorporating algae into layer diets [15]. Furthermore, storage time and conditions significantly impact egg quality, particularly Haugh units, with fresher eggs generally having higher quality. Lower or higher temperatures during storage can negatively affect egg quality, leading to deterioration.

Egg quality, defined by physical characteristics, is a critical attribute for consideration in retail due to its impact on consumer preferences, shelf-life, grading, and handling. As previously stated, the results of this study indicated high values for Haugh units and yolk index in all groups, which may be related to the use of very young animals and the short egg storage time. Regarding the egg shape index, Şekeroğlu and Duman [47] and Sekeroğlu et al. [38] found a strong positive correlation between the egg shape index and internal egg parameters (albumen length, yolk width, and yolk height). However, our findings did not observe the same pattern. Therefore, the higher egg shape index values in the 5 and 10% CV groups cannot be directly linked to an increase or decrease in these internal parameters. Further studies are needed to understand the reasons for the increase in egg shape index with the addition of algae to the diets of laying hens.

Overall, eggs produced by birds that received microalgae had lower shell index and thickness compared to eggs produced by the control group. Interestingly though, as egg weight increased when hens were fed 2.5% CV (as seen by the quadratic effect), the shell index and shell thickness were reduced at the same levels of CV incorporation. Ketta and Tumova [48] stated that eggshell quality can be affected by management conditions, genetics, age, oviposition time, and mineral nutrition. It is interesting to note that previous studies conducted by Kim and Kang [22] and Jeon et al. [49] found that the shell thickness of laying hens’ eggs was not significantly reduced, regardless of the concentration of CV added to their diet. Since animals had the same housing conditions and were of the same lineage and age, the results of eggshell parameters may be related to low mineral utilization in the experimental diet. However, further studies are needed to evaluate nutrient digestibility in chickens fed CV, as the calcium and phosphorus values reported in the literature show apparently similar values in corn, soybeans, and *Chlorella* sp. [50,51]. The absence of a significant impact on egg specific gravity suggests that adding microalgae to the hens’ diet did not affect the relative amount of eggshell compared to other components. This means that the structural integrity of the eggshell was maintained, and the risk of cracks during processing was not significantly altered [28]. Although there are limited data on pH analysis in eggs produced by hens that received CV in their diet, our findings align with the expectation that yolk pH values are lower than those of the albumen due to the presence of acidic components like phosphoric acid and citric acid in the yolk.

In the CV-treated groups, there was a significant improvement in yolk color, which can be attributed to the carotenoids present in the diet. These compounds are absorbed by the eggs from the feed and undergo metabolic transformations, ultimately leading to their accumulation in the yolk and the subsequent change in yolk color [52]. The carotenoids in the yolk also exhibit antioxidant properties, helping to protect lipids from oxidation [53].

Overall, previous studies on the incorporation of *Chlorella* in the diets of laying hens have revealed quite contradictory results. For instance, Kim and Kang. [22] found that adding 5% and 7.5% CV to diets significantly increased yolk color and Haugh units but did not affect shell thickness. On the other hand, Grigorova [23] observed that adding 10% of *Chlorella* to the diet negatively affected the shape index, significantly improved yolk color, and showed no significant differences in shell thickness.

At low concentrations, Englmaierová et al. [54] did not observe significant differences in Haugh units, albumen index, and yolk index when 1.25 g/kg of *Chlorella* was added to the diet of ISA Brown hens aged between 25 and 39 weeks, but they found that the egg surface area was larger in the group that received the algae. Similarly, Zheng et al. [13] noted that incorporating *Chlorella vulgaris* (1% and 2%) significantly improved Haugh units but did not alter shell thickness. Jeon et al. [49] found that Haugh units were highest in hens fed the diet containing 2.4% *Chlorella* and lowest in the control group, while shell thickness was not influenced. Kim et al. [55] observed that Haugh units and shell thickness were not affected by the diet (0.05% *Chlorella vulgaris*).

Studies involving the incorporation of other microalgae have also reported controversial results, but all of them have noted an increase in yolk color intensity [37,43,44]. Abbas et al. [43] also found that eggs produced by hens receiving *Spirulina platensis* in their diet (3%, 6%, 9%, and 12%) exhibited improved Haugh units and shell thickness. Tufarelli et al. [44] added 1% and 2% of *Spirulina platensis* and found no changes in shape index and Haugh unit. Omri et al. [37] reported a significant improvement in Haugh units when hens were supplemented with 1.5% and 2.5% of *Spirulina platensis* and a significant improvement in shell thickness only with 1.5% supplementation, with no significant differences observed at 2.5%. These authors also found that the yolk index remained unchanged with *Spirulina platensis* supplementation. Overall, our results demonstrate that dietary CV significantly influences several key egg quality parameters, particularly Haugh units, egg surface area, shape index, albumen index, and shell quality. While certain levels of CV may increase specific aspects of egg quality such as shape index and Haugh units, higher levels may negatively impact shell quality, indicating the need for careful consideration of the optimal CV concentration in laying hen diets.

## 5. Conclusions

Chlorella supplementation can positively influence egg quality and yolk color, though optimal levels are necessary to avoid negative effects on other performance parameters. The inclusion of CV in the diet resulted in a more orange-colored yolk. Therefore, CV has the potential to be an excellent natural pigment, thus reducing the addition of synthetic pigments to birds’ diets. In addition, the successful incorporation of 10% CV in substitution of SBM into laying hen diets indicates a significant advancement in reducing the reliance on inputs sourced from other continents, as well as a more sustainable environmental footprint associated with feed ingredient production, thereby fostering sustainability within the poultry industry. Long-term studies involving CV incorporation of greater than 2.5% in laying hen diets of varying ages are recommended to further investigate and clarify the effects on the production performance and quality of eggs. Future research should focus on a broader range of strains and housing conditions, as current studies primarily involve caged laying hens. Additionally, investigating the use of enzymes and extrusion to break down the cell wall of CV is crucial for optimizing its utilization in poultry diets. Further research is thus warranted to explore optimizing treatment levels to maximize beneficial outcomes, such as improved egg weight, without negatively impacting overall production and egg quality.

## Figures and Tables

**Table 1:** Composition of laying hens’ experimental diets.

	19–25 Weeks				26–34 Weeks			
**Ingredient (%)**	**Control**	**CV-2.5%**	**CV-5%**	**CV-10%**	**Control**	**CV-2.5%**	**CV-5%**	**CV-10%**
Corn	52.1	54.6	56.1	58.6	49.4	51.4	53.0	55.4
Soybean meal	30.5	27.0	23.7	16.5	32.1	28.8	25.4	18.3
Sunflower meal	1.60	1.50	1.52	2.00	1.60	1.54	1.54	2.10
Wheat	2.00	1.50	1.50	2.00	2.00	1.50	1.50	1.54
*Chlorella vulgaris*	0.00	2.50	5.00	10.0	0.00	2.50	5.00	10.0
Premix *	0.400	0.400	0.400	0.400	0.400	0.400	0.400	0.400
Salt	0.330	0.330	0.330	0.330	0.330	0.330	0.330	0.570
Sunflower oil	2.80	2.00	1.30	0.000	3.20	2.56	1.90	0.800
DL-Methionine	0.000	0.000	0.000	0.000	0.020	0.030	0.330	0.050
L-Lysine	0.010	0.010	0.020	0.020	0.000	0.000	0.000	0.000
Calcium carbonate	8.85	8.91	8.98	9.11	9.47	9.57	9.64	9.75
Dicalcium phosphate	1.38	1.30	1.20	1.00	1.50	1.39	1.29	1.10
**Calculated analyses**	**Control**	**CV-2.5%**	**CV-5%**	**CV-10%**	**Control**	**CV-2.5%**	**CV-5%**	**CV-10%**
Gross energy (cal/g)	2869	2866	2864	2866	2850	2853	2857	2858
Crude protein (%)	17.0	17.0	17.1	17.1	17.5	17.5	17.5	17.5
Methionine (%)	0.320	0.310	0.320	0.310	0.340	0.340	0.340	0.340
Lysine (%)	0.980	0.930	0.880	0.780	1.02	0.970	0.920	0.820
Calcium (%)	3.73	3.73	3.73	3.73	4.00	4.00	4.00	4.00
Phosphorus (%)	0.370	0.370	0.370	0.370	0.390	0.390	0.390	0.390
Sodium (%)	0.160	0.160	0.160	0.160	0.160	0.160	0.160	0.250
**Determined analyses**	**Control**	**CV-2.5**	**CV-5**	**CV-10**	**Control**	**CV-2.5**	**CV-5**	**CV-10**
Gross energy (cal/g)	3679	3670	3633	3620	3614	3634	3562	3555
Crude protein (%)	20.1	19.3	19.4	19.1	19.9	20.1	19.7	20.0
Dry matter (%)	89.9	89.9	90.0	90.2	90.0	90.0	90.2	90.3

Control: corn and soybean meal without CV; CV-2.5: diet with 2.5% CV; CV-5: diet with 5% CV; CV-10: diet with 10% CV. * Premix provided the following per kilogram of diet: vitamin A 25,000,000 UI, vitamin D3 625,000 UI, vitamin E 3750 mg, vitamin B1 250 mg, vitamin B2 1000 mg, vitamin B12 3 mg, vitamin K3 375 mg, D-calcium pantothenate 2000 mg, niacinamide 6250 mg, folic acid 62.5, choline chloride 75,000 mg, Cu 1750 mg, Zn 16,250 mg, Mn 21,250 mg, Fe 6250, Se 50 mg.

**Table 2:** Chemical composition of CV (dry matter basis).

Nutrients	Value (%)
Dry matter	93.1
Ash	8.70
Crude protein	55.2
Crude fat	9.40
Gross energy	3527 cal/g

**Table 3:** Effect of dietary CV on the productive performance of laying hens from 19 to 34 weeks of age.

Parameters	Treatment Groups				SEM	*p*-Value	
	Control	CV-2.5	CV-5	CV-10		Linear	Quadratic
Initial body weight (g)	1500	1460	1540	1480	0.019	0.615	0.981
Final body weight (g)	1820	1830	1740	1810	0.023	0.499	0.826
Egg production (%)	98.6	96.9	97.5	98.6	0.341	0.454	0.224
Egg weight (g)	60.9 ^ab^	62.2 ^a^	61.9 ^ab^	60.7 ^b^	0.204	0.019	0.007
Feed intake (g/d)	124 ^a^	119 ^a^	121 ^a^	118 ^b^	0.828	0.051	0.077
Feed conversion ratio	2.04 ^a^	1.94 ^b^	1.96 ^ab^	1.93 ^b^	0.015	0.100	0.081

Means within the same row with different superscripts are significantly different (*p* < 0.05). SEM: pooled standard error of the mean. Control: corn and soybean meal without CV. CV-2.5: diet with 2.5% CV. CV-5: diet with 5% CV. CV-10: diet with 10% CV.

**Table 4:** Effect of dietary CV on the egg quality parameters of laying hens from 19 to 34 weeks of age.

Parameters	Treatment Groups				SEM	*p*-Value	
	Control	CV-2.5	CV-5	CV-10		Linear	Quadratic
Haugh unit	86.6 ^ab^	84.6 ^b^	81.7 ^b^	90.7 ^a^	0.637	0.0003	0.0010
Egg surface area (cm^2^)	71.4 ^ab^	70.2 ^b^	72.8 ^a^	71.9 ^ab^	0.240	0.00080	0.828
Shape index (%)	78.5 ^c^	79.3 ^bc^	80.3 ^ab^	80.6 ^a^	0.161	<0.0001	<0.001
Yolk index (%)	48.3	47.8	47.9	49.8	0.292	0.114	0.0854
Albumen index (%)	7.39 ^ab^	7.50 ^ab^	6.65 ^b^	7.74 ^a^	0.127	0.017	0.166
Shell index (g/100cm^2^)	9.19 ^a^	8.41 ^b^	8.70 ^b^	8.67 ^b^	0.048	<0.0001	<0.0001
Shell thickness (mm)	0.391 ^a^	0.358 ^b^	0.365 ^b^	0.369 ^b^	0.002	<0.0001	<0.0001
Specific gravity	1.08	1.08	1.08	1.08	0.0004	0.995	0.964
Yolk pH	6.82	6.52	6.93	7.01	0.097	0.851	0.446
Albumen pH	9.11	8.78	10.2	8.64	0.290	0.230	0.668

Means within the same row with different superscripts are significantly different (*p* < 0.05). SEM: pooled standard error of the mean; Control: corn and soybean meal without CV; CV-2.5: diet with 2.5% CV; CV-5: diet with 5% CV; CV-10: diet with 10% CV.

**Table 5:** Egg yolk color from 19 to 34 weeks of age.

Parameters	Treatment Groups				SEM	*p*-Value	
	Control	CV-2.5	CV-5	CV-10		Linear	Quadratic
Roche/DSM	6.01 ^c^	7.43 ^b^	8.70 ^a^	9.15 ^a^	0.184	<0.0001	<0.0001
L*	42.6 ^a^	40.5 ^bc^	41.6 ^ab^	39.7 ^c^	0.169	<0.0001	<0.0001
b*	36.2 ^d^	38.6 ^c^	42.3 ^a^	41.1 ^b^	0.165	<0.0001	<0.0001
a*	1.73 ^d^	3.11 ^c^	5.15 ^b^	6.28 ^a^	0.0710	<0.0001	<0.0001

Means within the same row with different superscripts are significantly different (*p* < 0.05). SEM: pooled standard error of the mean; Control: corn and soybean meal without CV; CV-2.5: diet with 2.5% CV; CV-5: diet with 5% CV; CV-10: diet with 10% CV.

**Table 6:** Effects of diets on translucency.

Score	Treatment Groups				SEM	*p*-Value	
	Control	CV-2.5	CV-5	CV-10		Linear	Quadratic
Score 1	62.0	61.0	60.0	60.0	0.226	0.410	0.204
Score 2	25.0	25.0	27.0	27.0	0.707	0.831	0.618
Score 3	10.0	10.0	9.00	9.00	0.462	0.846	0.699
Score 4	3.00	4.00	4.00	4.00	0.182	0.803	0.811

SEM: pooled standard error of the mean; Control: corn and soybean meal without CV; CV-2.5: diet with 2.5% CV; CV-5: diet with 5% CV; CV-10: diet with 10% CV.

## Data Availability

The data supporting the findings of this study are available from the authors upon request.
